# Incidence and Progression of Echocardiographic Abnormalities in Older Children with Human Immunodeficiency Virus and Adolescents Taking Antiretroviral Therapy: A Prospective Cohort Study

**DOI:** 10.1093/cid/ciz373

**Published:** 2019-05-04

**Authors:** Edith D Majonga, Andrea M Rehman, Grace Mchugh, Hilda A Mujuru, Kusum Nathoo, Jon O Odland, Rashida A Ferrand, Juan Pablo Kaski

**Affiliations:** 1 London School of Hygiene and Tropical Medicine, United Kingdom; 2 Biomedical Research and Training Institute, Harare; 3 University of Zimbabwe, Harare; 4 The Norwegian University for Science and Technology, Trondheim; 5 Department of Public Health, University of Pretoria, South Africa; 6 Centre for Inherited Cardiovascular Diseases, Great Ormond Street Hospital; 7 Institute of Cardiovascular Science, University College London, United Kingdom

**Keywords:** cardiac abnormalities, HIV, children, ART, echocardiography

## Abstract

**Background:**

A high prevalence of cardiac abnormalities has been reported in children with human immunodeficiency virus (HIV) taking antiretroviral therapy (ART) in sub-Saharan Africa. We investigated the incidence and progression of cardiac abnormalities among children taking ART in Zimbabwe.

**Methods:**

A prospective cohort study was conducted at a pediatric HIV clinic from 2014 to 2017. Children with HIV aged between 6 and 16 years and taking ART ≥6 months were enrolled. Transthoracic echocardiography was performed at baseline and after 18 months.

**Results:**

Of 197 participants recruited at baseline, 175 (89%; 48% female; median age 12 years, interquartile range 10–14 years) were followed up. The incidences of left and right heart abnormalities were 3.52 and 5.64 per 100 person-years, respectively. Stunting was associated with the development of any cardiac abnormality (adjusted odds ratio 2.59, 95% confidence interval 1.03–6.49; *P* = .043). Right ventricular (RV) dilatation persisted at follow-up in 92% of participants and left ventricular (LV) diastolic dysfunction in 88%. Cardiac abnormalities present at baseline reverted to normal over the follow-up period in 11 (6%). There was an overall increase in mean z scores for LV, left atrium (LA), RV, interventricular septum, and LV posterior wall diameters at 18 months (*P* < .001).

**Conclusions:**

Despite ART, children with HIV have a high incidence of cardiac abnormalities, with only a minority being transient. Mean z scores for LV, LA, RV, interventricular septum, and LV posterior wall diameters increased over a relatively short follow-up period, suggesting the potential for progression of cardiac abnormalities. Longer follow-up is required to understand the clinical implications of these abnormalities.

## Teaser

Older children and adolescents with human immunodeficiency virus (HIV) who are receiving antiretroviral therapy in sub-Saharan Africa have a high incidence of echocardiographic abnormalities, which are likely primary HIV-related heart muscle disease and have the potential for progression over time.

The global scale-up of antiretroviral therapy (ART) programs has been followed by a dramatic decline in mortality among individuals living with human immunodeficiency virus (HIV) infection [1]. While ART facilitates immune reconstitution and reduces the risk of infections, there is increased recognition that a long-standing HIV infection is associated with an increased risk of chronic comorbidities. This may be a result of the HIV infection itself, its treatment, or sequelae of infections [2]. One of the most well-recognized comorbidities is cardiac disease, with several studies showing an increased risk of adults developing cardiac disease despite ART [3, 4]. An important limitation of studies in adults is confounding by well-established risk factors for cardiac disease, such as age, smoking, and hypertension [4].

In the pre-ART era, infants and younger children with HIV were also reported to be at increased risk of developing cardiac disease, with a 5-year cumulative incidence of cardiac dysfunction of 18–39% [5]. The most commonly reported abnormalities were left ventricular (LV) systolic dysfunction and LV dilatation; these were often progressive and were a predictor of all-cause mortality [6, 7]. Most of these studies were conducted in high-income settings, mainly in younger children, and with some cohorts including children taking monotherapy for the treatment of an HIV infection [5, 7]. In high-income countries, most children start ART in infancy, which not only decreases mortality but may prevent organ damage [8]; in these settings, the incidence of cardiac disease in children on ART has declined [9]. These findings cannot be generalized to children growing up with HIV in sub-Saharan Africa (SSA), where 90% of the world’s children with HIV live, most of whom have had delayed diagnoses of HIV and/or started ART in older childhood [10, 11].

Nevertheless, increasing numbers of children in SSA, who would have died in early childhood, are now reaching adolescence and adulthood due to ART [12]. We and others have reported high prevalences of cardiac abnormalities among African children, despite treatment with ART and the absence of the traditional risk factors for cardiac disease [13, 14]. However, little is known about the incidences and clinical courses of cardiac abnormalities in African children in the ART era. We investigated the incidences and progressions of cardiac abnormalities in children taking ART in Harare, Zimbabwe.

## METHODS

A prospective cohort study was conducted from August 2014 to December 2017 at the pediatric HIV clinic at Harare Central Hospital, Zimbabwe, a public-sector HIV clinic that provides care to over 4000 children. ART is provided free of charge, according to national guidelines. This study was part of a larger study aiming to investigate cardiorespiratory diseases in children with HIV infections who are taking ART (Investigation of Heart and Lung Diseases in HIV Among Older Children; INHALE). Baseline cardiac findings in this cohort of HIV-infected children have recently been reported [13]. Findings from this cohort pertaining to chronic lung disease have also been published [15, 16].

### Participants

Children with HIV aged 6 to 16 years, attending the outpatient HIV clinic, taking ART for at least 6 months, and clinically stable (defined as not requiring hospital admission and not too ill to participate) were consecutively enrolled on weekdays, limited to the first 5 eligible participants per day for logistical ease, as previously described [13]. Participants were followed up at 18 months.

### Study Procedures

An interviewer-administered questionnaire was used to collect sociodemographic data and clinical histories. Assessments of pubertal stage were based on Tanner staging [17]. Each clinical assessment included measurements of height, weight, heart and respiratory rates and blood pressure, pulse oximetry, spirometry, HIV viral load, and CD4 count tests and a transthoracic echocardiogram (full methods in Supplementary Data 1). The methods and definitions of this study have also been previously described [13].

### Data Management and Statistical Analysis

Data were extracted from paper forms using optical character recognition software (Cardiff Teleform Intelligent Character, Version 10.7) and analyzed using STATA version 12 software (StataCorp, TX). Continuous data were presented as means ± standard deviations (SDs) if they were normally distributed or medians (interquartile ranges, IQRs) if not normally distributed. Paired *t*, Mann-Whitney, and McNemar tests were used to compare clinical characteristics of participants and mean values of cardiac measures and z scores at baseline and follow-up. There was temporal blinding during an analysis of echocardiograms at follow-up. The mean change in z scores for each cardiac measure was calculated as mean z score at 18 months minus mean z score at baseline, and adjusted for baseline z scores using linear regression. The incidence rates and risks for right and left heart abnormalities were calculated in those without abnormalities at baseline, and risk is reported as a proportion. Logistic regression was used to assess for risk factors for incident cardiac abnormalities at follow-up. These included baseline HIV-related and clinical factors. As previously described, HIV-related factors were categorized as CD4 cell count (>200 cells/µl and ≤200 cells/µl), HIV viral load (≤400 copies/µl and >400 copies/µl), age at ART initiation (0–5, 6–10, and 11–16 years), and duration on ART (≤2 years and >2 years) [13]. Age (categorized as 6–10 years and 11–16 years) and sex were included as *a priori* variables. Antiretroviral drugs, including nevirapine and zidovudine, were also assessed. HIV-related variables with *P* values ≤ 0.1 were retained for inclusion in the multivariate model; clinical factors were added into the model individually, and those which were significant at *P* ≤ 0.1 were retained for inclusion in the final model. We explored the association between baseline factors as linear variables and mean changes in z scores for cardiac measures, adjusted for baseline z scores using linear regression. A *P* value ≤0.05 was considered statistically significant.

Ethical approval was obtained from the Medical Research Council of Zimbabwe, the London School of Hygiene and Tropical Medicine Ethics Committee, the Biomedical Research and Training Institute Institutional Review Board, and the Harare Central Hospital Ethics Committee. Written informed consent from guardians and assent from participants were obtained prior to enrollment.

## RESULTS

### Clinical Characteristics

**Figure 1.**
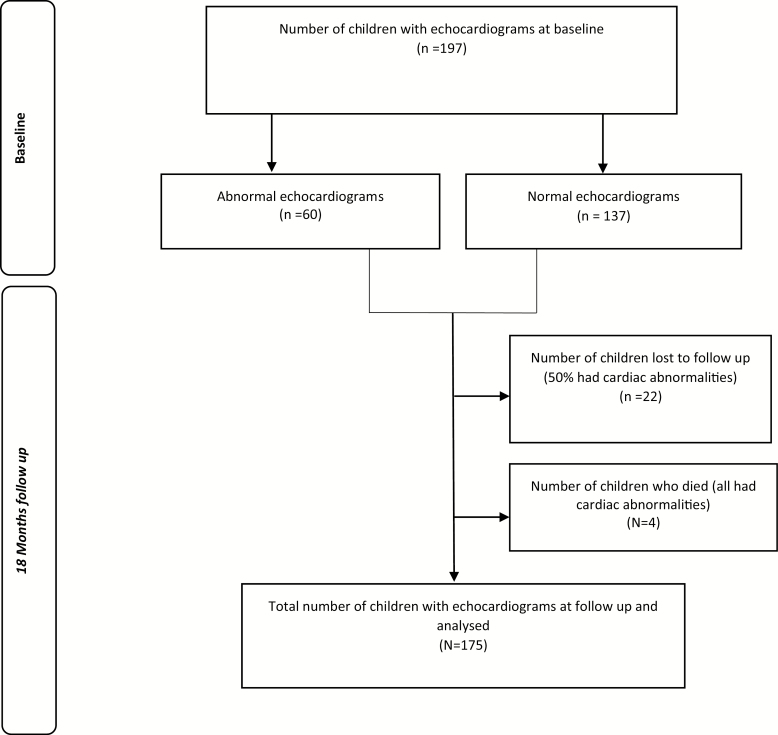
Flow chart for participant recruitment and follow-up.

A total of 197 participants were recruited, of whom 175 (89%) were followed up at 18 months, giving 283.9 person-years (pys) of follow-up (Figure 1). The median age of participants at the 18 month follow-up was 12 years (IQR 10–14 years), and 84 (48%) were female. At 18 months, 40% of participants were virally suppressed (<400 copies/ml), compared to 78% at baseline (*P* < .001; Table 1). Supplementary Figure 1 shows the flow diagram of viral suppression over time. There were 4 participants who died, all of whom had echocardiographic abnormalities at baseline. The causes of death were pulmonary tuberculosis; meningitis; cardiac failure due to dilated cardiomyopathy; and unknown. The 22 participants lost to follow-up either relocated or were unreachable due to changed contact details. There were no significant differences in age, CD4 count, viral load, duration on ART, clinical characteristics, height-for-age and weight-for-age z scores, and cardiac dimension z scores at baseline between participants who were followed up and those lost to follow-up. Data on pubertal staging were available at follow-up on all participants. There were 30 (35%) participants who had reached menarche. Of the female participants, 34 (40%) and 40 (48%) were in Tanner Stage 1 of breast and pubic hair development, respectively, compared to 3 (3%) testicular volume and 66 (73%) pubic hair development of the male participants (data not shown).

**Table 1.** Clinical Characteristics of Participants

Variable	(N = 175)		*P* Value
	Baseline Mean (SD)	18 Months Mean (SD)	
Female, n (%)	84 (48)		…
Age, years, median (IQR)	11 (9–13)	12 (10–14)	**<.001**
CD4, cell/μl, median (IQR)	726 (473–935)	734 (462–989)^a^	.455
Viral load, copies/ml, median (IQR)	19 (19–208)	456 (165–4080)^b^	**<.001**
Duration on ART, years, median (IQR)	4.8 (2.8–6.4)	6.5 (4.3–8.1)	**<.001**
Systolic blood pressure, mmHg	110 (11)	109 (10)	.247
Diastolic blood pressure, mmHg	73 (9)	72 (9)	.392
Respiratory rate, breaths per min	21.8 (4.5)	21.9 (2.3)	.697
Signs and symptoms, n (%)			
Chest pains on exertion	20 (11)	5 (3)	**<.001**
Tachycardia	10 (6)	6 (3)	.317
Tachypnoea	24 (14)	10 (6)	**.013**
Hypoxia at rest	1 (1)	0	…
Abnormal spirometry^c^	37 (24)	23 (15)	.144
Wasting	38 (22)	35 (20)	.106
Stunting	40 (23)	39 (22)	.117
Cardiac measures			
RV diameter z score	0.40 (1.3)	0.91 (1.1)	**<.001**
LV diameter z score	0.49 (1.1)	0.72 (1.1)	**<.001**
IVS diameter z score	0.06 (1.0)	0.65 (0.8)	**<.001**
LVPW diameter z score	0.29 (1.2)	0.88 (0.9)	**<.001**
LA diameter z score	0.36 (1.1)	0.66 (1.0)	**<.001**
TAPSE	–0.63 (0.9)	–0.26 (1.0)	**<.001**
Ejection fraction (%)	61.7 (6.2)	64.5 (6.6)	**<.001**
E wave (m/s)	0.91 (0.1)	0.93 (0.2)	.142
A wave (m/s)	0.53 (0.1)	0.55 (0.1)	.105
E/A ratio	1.76 (0.4)	1.74 (0.4)	.656
Deceleration time (ms)	174 (27.7)	169 (15.6)	**.044**
PV S wave (m/s)	0.49 (0.1)	0.51 (0.1)	.069
PV D wave (m/s)	0.55 (0.5)	0.51 (0.1)	.388
PV A wave (m/s)	0.19 (0.2)	0.22 (0.5)	.352
PV S/D ratio	0.98 (0.3)	1.02 (0.2)	.062
Cardiac abnormalities, n (%)			
LV dilatation	7 (4)	8 (5)	.317
LVH	18 (10)	15 (9)	.257
LA dilatation	14 (8)	15 (9)	.317
LV systolic dysfunction	2 (1)	1 (1)	.563
LV diastolic dysfunction	40 (23)	40 (23)	1.000
RV dilatation	12 (7)	23 (13)	**.002**
RV systolic dysfunction	2 (1)	6 (3)	**.046**
Tricuspid regurgitation	31 (18)	34 (20)	.564
Mitral regurgitation	5 (3)	3 (2)	.317
Pulmonic regurgitation	37 (21)	28 (16)	**<.001**
Aortic regurgitation	…	…	…

A *P* value ≤.05 was considered statistically significant. Therefore, all bold values were statistically significant.

Abbreviations: A wave, late diastolic filling velocity; ART, antiretroviral therapy; D, diastolic; E wave, early diastolic filling velocity; E/A ratio, mitral valve peak early to late left ventricular filling velocity; IQR, interquartile range; IVS, interventricular septum; LA, left atrium; LV, left ventricle; LVH, left ventricular hypertrophy; LVPW, left ventricular posterior wall; PV, pulmonary venous; RV, right ventricle; S, systolic; SD, standard deviation; TAPSE, tricuspid annular plane systolic excursion.

^a^n = 159

^b^n = 91

^c^n = 150

### Echocardiographic Findings

Baseline echocardiographic findings have been previously reported [13]. Briefly, at baseline, 40 participants (23%) had LV diastolic dysfunction; 18 (10%) had left ventricular hypertrophy (LVH); 14 (8%) had left atrium (LA) dilatation; 2 (1%) had right ventricular (RV) systolic dysfunction; and 12 (7%) had RV dilatation. At 18 months, 23 (13%) participants had RV dilatation and 6 (3%) had RV systolic dysfunction. Apart from right heart abnormalities, there were no significant changes in the proportions of left heart cardiac abnormalities at follow-up (Table 1). We observed overall increases in mean z scores for LV, LA, RV, interventricular septum, and LV posterior wall diameters and tricuspid annular plane systolic excursion for all participants at follow-up (*P* < .001; Table 1). Table 2 shows mean baseline and 18-month z scores, adjusted mean (SD) changes in z scores for cardiac parameters, and estimated risks for cardiac abnormalities. The risk of developing RV dilatation was highest (12/163, 7%) with a mean (SD) z score for RV diameter at baseline of +1.10 (0.7), which increased to +2.51 (0.5). Of those with RV dilatation at follow-up, 4 had mild tricuspid regurgitation and 7 had mild pulmonary regurgitation, 2 of whom had both tricuspid and pulmonary regurgitation. There was a negative correlation between the change in z score and baseline z scores: that is, participants with high z scores at baseline had smaller changes in z scores at follow-up. Mean changes in z scores, adjusted for baseline z scores of the same parameter, were 0.64 SD (95% confidence interval [CI] 0.52–0.77) for the RV diameter; 0.36 SD (95% CI 0.25–0.47) for the LV diameter; 0.63 SD (95% CI 0.52–0.74) for the interventricular septum diameter; 0.74 SD (95% CI 0.63–0.85) for the LV posterior wall diameter; 0.42 SD (95% CI 0.33–0.51) for the LA diameter; and –0.06 SD (95% CI –0.24 to 0.13) for tricuspid annular plane systolic excursion. Overall, 10 participants developed left heart abnormalities and 16 developed right heart abnormalities at 18 months, giving estimated incidences of 3.52/100 pys and 5.64/100 pys, respectively.

**Table 2.** Z Scores at Baseline and Follow-up for Participants

Cardiac Variable	Baseline	Follow-up	N (%)	Z Score Baseline Mean (SD)	Z Score 18 Months Mean (SD)	Adjusted Change in Z Score after 18 Months, SD (95% CI)	*P* Value	Risk
RV diameter diastole	Normal	Normal	151 (86)	0.15 (1.1)	0.64 (0.9)	0.55 (.43–.67)	<.001	…
	Normal	RV dilatation	12 (7)	1.10 (0.7)	2.51 (0.5)	2.47 (1.90–3.03)	<.001	12/163 (7%)
	RV dilatation	Normal	1 (1)	2.23 (0.0)	1.81 (0.0)	–0.79^a^	…	…
	RV dilatation	RV dilatation	11 (6)	2.91 (0.7)	2.85 (0.8)	0.59 (–1.21 to 2.39)	.478	…
LV diameter diastole	Normal	Normal	167 (95)	0.37 (1.0)	0.62 (1.0)	0.35 (.24–.47)	<.001	…
	Normal	LV dilatation	1 (1)	1.04	2.10	1.06^a^	…	1/168 (1%)
	LV dilatation	LV dilatation	7 (4)	3.27 (0.3)	2.94 (0.4)	–0.46 (–2.20 to 1.27)	.523	…
IVS diameter diastole	Normal	Normal	172 (98)	0.02 (0.9)	0.62 (0.8)	0.61 (.51–.71)	<.001	…
	Normal	IVS hypertrophy	2 (1)	1.82 (0.2)	3.13 (1.1)	1.31 (1.3)	…	2/174 (1%)
	IVS hypertrophy	Normal	1 (1)	2.50	1.63	–0.87^a^	…	…
LVPW diameter diastole	Normal	Normal	158 (90)	0.05 (1.0)	0.72 (0.8)	0.70 (.59–.81)	<.001	…
	LVPW hypertrophy	Normal	4 (2)	2.43 (0.2)	1.63 (0.2)	–0.80 (.4)^a^	…	…
	LVPW hypertrophy	LVPW hypertrophy	13 (7)	2.61 (0.5)	2.61 (0.4)	1.00 (.11–1.89)	.032	…
LA diameter diastole	Normal	Normal	160 (91)	0.17 (1.0)	0.52 (0.9)	0.41 (.31–.50)	<.001	…
	Normal	LA dilatation	1 (1)	1.99	2.07	0.08^a^	…	1/161 (1%)
	LA dilatation	LA dilatation	14 (8)	2.44 (0.3)	2.44 (0.2)	2.74 (1.77–3.70)	<.001	…
TAPSE	Normal	Normal	154 (88)	–0.59 (0.9)	–0.16 (1.0)	–0.04 (.22–.14)	.673	…
	Normal	RV systolic dysfunction	4 (2)	–1.36 (0.6)	–2.32 (0.3)	–0.96 (.8)^a^	…	4/158 (3%)
	RV systolic dysfunction	RV systolic dysfunction	2 (1)	–2.23 (0.1)	–2.20 (0.1)	–0.02 (.1)^a^	…	…

Abbreviations: CI, confidence interval; IVS, interventricular septum; LA, left atrium; LV, left ventricle; LVPW, left ventricular posterior wall; RV, right ventricle; SD, standard deviation; TAPSE, tricuspid annular plane systolic excursion.

^a^Unadjusted change (if n = 1) or adjusted mean (and SD if n > 1) has been reported.

Of the 23 (13%) participants who had RV dilatation at follow-up, 10 (43%) had concurrent left heart abnormalities and 11 (48%) also had RV dilation at baseline. Of the 6 participants with RV systolic dysfunction at 18 months, 2 had systolic dysfunction at baseline. None of the participants met the criteria for pulmonary hypertension by echocardiography. LVH was present in 15 (9%) participants at follow-up, of whom 13 (87%) also had LVH at baseline and 3 (20%) and 5 (33%) had LA dilatation and LV diastolic dysfunction, respectively, at baseline. LV dilatation persisted from baseline in 7 (4%) participants, 2 of whom had concurrent LA dilatation at baseline and follow-up. Regression of abnormalities was observed in 11 (6%) participants: 1 had LV systolic dysfunction, 3 had LVH, 1 had LVH with concurrent LV systolic dysfunction, 4 had isolated LV diastolic dysfunction, 1 had isolated LV diastolic dysfunction with concurrent LVH, and 1 had RV dilatation at baseline. Cardiorespiratory symptoms were reported by 48/175 (27%) at baseline and, of these, 85% were asymptomatic at 18 months.

On multivariate logistic regression, stunting was associated with the development of any new cardiac abnormality (adjusted odds ratio 2.59, 95% CI 1.03–6.49; *P* = .043). No HIV-related factors, including CD4 count, viral load, duration on ART, age at ART initiation, and type of ART, were associated with incidences of any cardiac abnormalities. Supplementary Table 1 shows the types of ART and proportions of participants receiving the different drugs. There were no associations between incidences of right heart abnormalities and abnormal spirometry. Menarche or the different indices of pubertal growth were not associated with changes in mean z scores for cardiac dimensions (data not shown). On linear regression, no association was observed between baseline factors and mean changes in z scores for cardiac dimensions (Supplementary Table 2).

## DISCUSSION

To our knowledge, this is the first cohort study from SSA reporting the incidences and progressions of cardiac abnormalities in children taking ART. Despite ART, participants continued to develop cardiac abnormalities, with the highest risk being development of RV dilatation, which was not associated with abnormal lung functions. Most of the children with RV dilatation had isolated dilatation without associated RV systolic dysfunction. Currie et al [18]reported that heart muscle disease among people with HIV manifests as global or borderline LV dysfunction and isolated RV dilatation; we hypothesize that the isolated RV dilatation observed in the present study may be part of the spectrum of HIV-related cardiomyopathy, and that it manifests before LV involvement becomes apparent. Isolated RV enlargement has been previously reported in adults with HIV who had preserved LV functions [19]. Notably, none of the participants with RV dilatation met the criteria for pulmonary hypertension using Doppler echocardiography, although we acknowledge that right heart cardiac catheterization is the gold standard in diagnosing pulmonary hypertension. Despite the limitations of Doppler echocardiography in assessing right heart pressures in children, there was no association between right heart abnormalities and abnormal lung functions, further suggesting that the abnormalities are more likely due to primary heart muscle disease.

Most cardiac abnormalities present at baseline persisted at follow-up, and we observed overall increases in mean z scores for cardiac dimensions over this short period of follow-up. However, the majority had no symptoms, suggesting that there may be a prolonged period of subclinical cardiac abnormalities before overt disease develops. The results of our study suggest the potential for the progression of cardiac abnormalities and highlight the importance of routine screening for cardiac disease in children with HIV, even in the absence of symptoms.

A minority of participants had transient cardiac abnormalities. LV systolic dysfunction and LVH may have been consequences of acute infectious myocarditis [20]. Transient ventricular wall thickening has previously been observed in participants with acute myocarditis [21]. The participant with RV dilatation at baseline that resolved by follow-up had a respiratory tract infection near the time of the initial echocardiogram, which then resolved. A lung infection may result in transient pulmonary hypertension and RV enlargement, which resolve with effective treatment of the infection [22]. Reversible cardiac abnormalities have also been reported among HIV-infected adults in the United States, more than two-thirds (71%) of whom had acquired immunodeficiency syndrome [23].

The etiology of myocardial disease in HIV infections is complex. Several pathogenic mechanisms have been hypothesized, including the chronic systemic immune activation of cardiac myocytes that occurs in HIV infection and is not completely reversed by ART [24]. Infection of the heart by opportunistic pathogens, including cytomegalovirus, Epstein Barr virus, coxsackievirus, and adenovirus, may also result in cardiac damage [25]. Cardiotoxicity from ART has also been suggested, particularly in nucleoside reverse transcriptase inhibitors, the backbone of ART, including abacavir and zidovudine [26, 27]. This may be related to mitochondrial dysfunction, which has been observed following the use of stavudine, didanosine, and zidovudine, while abacavir reportedly increases the risk of myocardial infarction in adults [28]. Abacavir and zidovudine were being taken by 4% and 52% of the participants, respectively, but no associations between antiretroviral drugs and cardiac abnormalities were identified in this study. This further supports the possibility of the abnormalities being due to primary heart muscle disease.

Stunted children had a higher likelihood of developing a cardiac abnormality. Miller et al [29]also reported that stunting was associated with LV diastolic dysfunction in their retrospective study of HIV-infected adolescents, 71% of whom were on ART. Stunting is a marker of chronic inflammatory conditions in childhood, and underlying systemic inflammation may also play a role in the pathogenesis of cardiac disease [30, 31]. Notably, the proportion of participants who were virally nonsuppressed had increased over the follow-up period. Poor adherence and high rates of viral nonsuppression among adolescents have been reported previously [32]. This would lead to ongoing viral replication, which is, in turn, associated with dysregulated systemic immune activation. In adults with HIV, systemic immune activation is a risk factor for the development of cardiac disease [30]. HIV persists in reservoir cells even after effective treatment with ART and may continue to release cytotoxic cytokines, which subsequently contribute to progressive and late tissue damage (cardiac myocytes) [33]. The median age of ART initiation in this cohort was 6 years (IQR 3–8 years), and immunosuppression and opportunistic infections prior to ART initiation may cause cardiac damage. Poor adherence to ART may also explain the higher incidence of cardiac disease in our cohort, compared to previous studies in high-income settings [9].

### Strengths and Limitations

This study is the first to report the incidence and progression of cardiac disease in a pediatric HIV population from SSA, where the vast majority of children with HIV live. The main strengths include the prospective, systematic evaluation for cardiac abnormalities and the use of local reference ranges to define the abnormalities, rather than reference ranges derived from North American or European populations, which have been shown to be inappropriate for SSA [34, 35]. Furthermore, participants were consecutively enrolled and not selectively enrolled on the basis of symptoms. Limitations of the study include the lack of viral load measures on all participants and, as a result, we may have been underpowered to detect any associations between incident cardiac diseases and viral loads. The study of biomarkers was not within the scope of this study, but we do have stored blood samples and the investigation of systemic markers of inflammation is ongoing. The use of advanced echocardiographic techniques, such as 3-dimensional echocardiography, may have provided more imaging by allowing for the direct calculation of chamber volumes, as well as global or regional functions through the elimination of geometric assumptions used in M-mode, and minimized the errors arising from foreshortened apical views. Notably, we only used optimum images to acquire the various measurements in this study. Speckle tracking imaging would have been more sensitive for detecting subclinical ventricular functions, which may not be identified by measurements of the LV ejection fraction. Tissue Doppler imaging may have assessed LV diastolic dysfunction better, because it is less dependent on loading conditions, compared to transmitral inflow velocities. However, we also included pulmonary venous flow velocities, which provided additive value to the evaluation of LV diastolic functions. Importantly, this study was performed in a resource-limited setting, and the use of more contemporary imaging measures was not available. In most healthcare settings in this region, there is not widespread availability of advanced echocardiographic techniques, and so our findings will be more readily applicable to low-resource settings, which is where the majority of these patients are seen.

## CONCLUSION

There are high incidences of cardiac abnormalities in children with HIV who are taking ART in SSA, and the lack of association with lung and HIV factors suggests that these abnormalities are primary HIV-related heart muscle disease. There is some evidence of disease progression over a short follow-up period. Longer follow-ups are needed to understand the clinical implications of these abnormalities, and the pathogenesis of these abnormalities needs further study.

## Supplementary Data

Supplementary materials are available at *Clinical Infectious Diseases* online. Consisting of data provided by the authors to benefit the reader, the posted materials are not copyedited and are the sole responsibility of the authors, so questions or comments should be addressed to the corresponding author.

ciz373_suppl_Supplementary_Data_1

ciz373_suppl_Supplementary_Figure_1

ciz373_suppl_Supplementary_Table_1

ciz373_suppl_Supplementary_Table_2

## Notes

***Acknowledgments.*** The authors thank Harare Children’s Hospital, the clinic staff, participants and their families, and the Human Immunodeficiency Virus Research Trust.

***Disclaimer.*** The views expressed are those of the author(s) and not necessarily those of the National Health Services, the National Institute for Health Research (NIHR), or the Department of Health.

***Financial support.*** This work was funded by the Wellcome Trust (grant number 095878/Z/11/Z), Nina-Ireland Program for Lung Health, and Norwegian Research Council through Global Health and Vaccination Research.

***Potential conflicts of interest.*** A. M. R. receives a salary from the United Kingdom Medical Research Council through a grant to the London School of Hygiene & Tropical Medicine Tropical Epidemiology Group (grant number MR/K012126/1). J. P. K. is a member of the European Reference Network on Heart Diseases (ERN GUARD-HEART) and is supported by Max’s Foundation, Great Ormond Street Hospital Children’s Charity, and the NIHR Great Ormond Street Hospital Biomedical Research Centre. All other authors report no potential conflicts. All authors have submitted the ICMJE Form for Disclosure of Potential Conflicts of Interest. Conflicts that the editors consider relevant to the content of the manuscript have been disclosed.
