# Supplementary material for: Incidence and Progression of Echocardiographic Abnormalities in Older Children with Human Immunodeficiency Virus and Adolescents Taking Antiretroviral Therapy: A Prospective Cohort Study
Source: Clin Infect Dis. 2019 May 4;70(7):1372–8. doi: 10.1093/cid/ciz373 (PMC7931829; doi:10.1093/cid/ciz373)
Supplement: ciz373_suppl_Supplementary_Data_1 [file ciz373_suppl_supplementary_data_1.docx]

**Supplementary Data 1**

**Methods**

Transthoracic echocardiography was performed by an echocardiographer trained in pediatric echocardiography (EDM). A standard protocol consisting of 2-dimensional, M-mode, pulsed and continuous wave Doppler as recommended by American Society of Echocardiography (ASE) was adopted for image acquisition and cardiac measurements [1] Images were acquired and saved in DICOM format for off-line analysis. Blood samples were collected for HIV viral load and CD4 count testing. All clinical assessments were repeated at 18-month follow up.

***Definitions***

The echocardiographic cardiac measures were normalized to body surface area (BSA) (calculated using the Du Bois and Du Bois method) [2] and converted to z-scores using previously-published local references [3]. Echocardiographic abnormalities were broadly categorized as right and left heart abnormalities. Right heart abnormalities refer to either right ventricular (RV) dilatation (defined as a z-score >+2 for RV diameter end-diastole); and/or systolic dysfunction [defined as a tricuspid annular plane systolic excursion (TAPSE) z-score of < -2]; and/or pulmonary hypertension [defined as present if the tricuspid regurgitation velocity was ≥2.9 m/s, pulmonary arterial systolic pressure (PASP) ≥37 mmHg with/without additional echocardiographic variables suggestive of pulmonary hypertension (assuming right atrial pressure of 5mmHg)[4]]. Left heart abnormalities included LV dilatation (defined as a z-score >+2 for LV end-diastolic diameter) or hypertrophy (defined as maximal wall thickness of interventricular septum (IVS) and/or LV posterior wall (LVPW) greater than +2 z-scores); and/or left atrial (LA) dilatation (defined as z-score >+2 for LA end-systolic diameter); and/or systolic and/or diastolic dysfunction. LV systolic function was assessed using Simpson’s Biplane method and an ejection fraction ≥55% was considered normal [1]. LV diastolic dysfunction was assessed using transmitral Doppler peak early (E) and late diastolic (A) filling velocities, E/A ratio, deceleration time and pulmonary venous flow velocities, including peak systolic (S) and diastolic (D) waves, S/D ratio and atrial reversal (Ar) velocity; paediatric reference ranges were used to define abnormality [5]. Participants were classified as having diastolic dysfunction when at least four parameters were abnormal.

Resting tachypnoea was defined as a respiratory rate >25/min. Stunting and wasting were defined as a z-score <-2 for height-for-age and weight-for-age respectively, using British 1990 growth references [6]. Hypoxia was defined as a resting oxygen saturation <88%. Abnormal spirometry was defined as a reduced ratio of the highest forced expiratory volume in 1 second (FEV1) and forced vital capacity (FVC) (FEV1: FVC) or reduced FVC regardless of normal FEV1: FVC ratio not reversed with salbutamol [7].

**Additional References**

1. Lopez L, Colan SD, Frommelt PC, et al. Recommendations for quantification methods during the performance of a pediatric echocardiogram: a report from the Pediatric Measurements Writing Group of the American Society of Echocardiography Pediatric and Congenital Heart Disease Council. *J Am Soc Echocardiogr* **2010**; 23:465-95; 576-7

2. Du Bois D, Du Bois, EF. A formula to estimate the approximate surface area if height and weight be known. 1916. *Nutrition (Burbank, Los Angeles County, Calif)* **1989**; 5:303

3. Majonga ED, Rehman AM, McHugh G, et al. Echocardiographic reference ranges in older children and adolescents in sub-Saharan Africa. *International Journal of Cardiology* **2017**; 248:409-13

4. Galie N, Hoeper MM, Humbert M, et al. Guidelines for the diagnosis and treatment of pulmonary hypertension: the Task Force for the Diagnosis and Treatment of Pulmonary Hypertension of the European Society of Cardiology (ESC) and the European Respiratory Society (ERS), endorsed by the International Society of Heart and Lung Transplantation (ISHLT). *Eur Heart J* **2009**; 30:2493-537

5. O'Leary PW, Durongpisitkul K, Cordes TM, et al. Diastolic ventricular function in children: a Doppler echocardiographic study establishing normal values and predictors of increased ventricular end-diastolic pressure. *Mayo Clin Proc* **1998**; 73:616-28

6. Cole TJ, Freeman JV, Preece MA. British 1990 growth reference centiles for weight, height, body mass index and head circumference fitted by maximum penalized likelihood. *Stat Med* **1998**; 17:407-29

7. Rylance J, McHugh G, Metcalfe J, et al. Chronic lung disease in HIV-infected children established on antiretroviral therapy. *AIDS* **2016**; 30:2795-803
