# Supplementary figures and images for: Incidence and Progression of Echocardiographic Abnormalities in Older Children with Human Immunodeficiency Virus and Adolescents Taking Antiretroviral Therapy: A Prospective Cohort Study
Source: Clin Infect Dis. 2019 May 4;70(7):1372–8. doi: 10.1093/cid/ciz373 (PMC7931829; doi:10.1093/cid/ciz373)

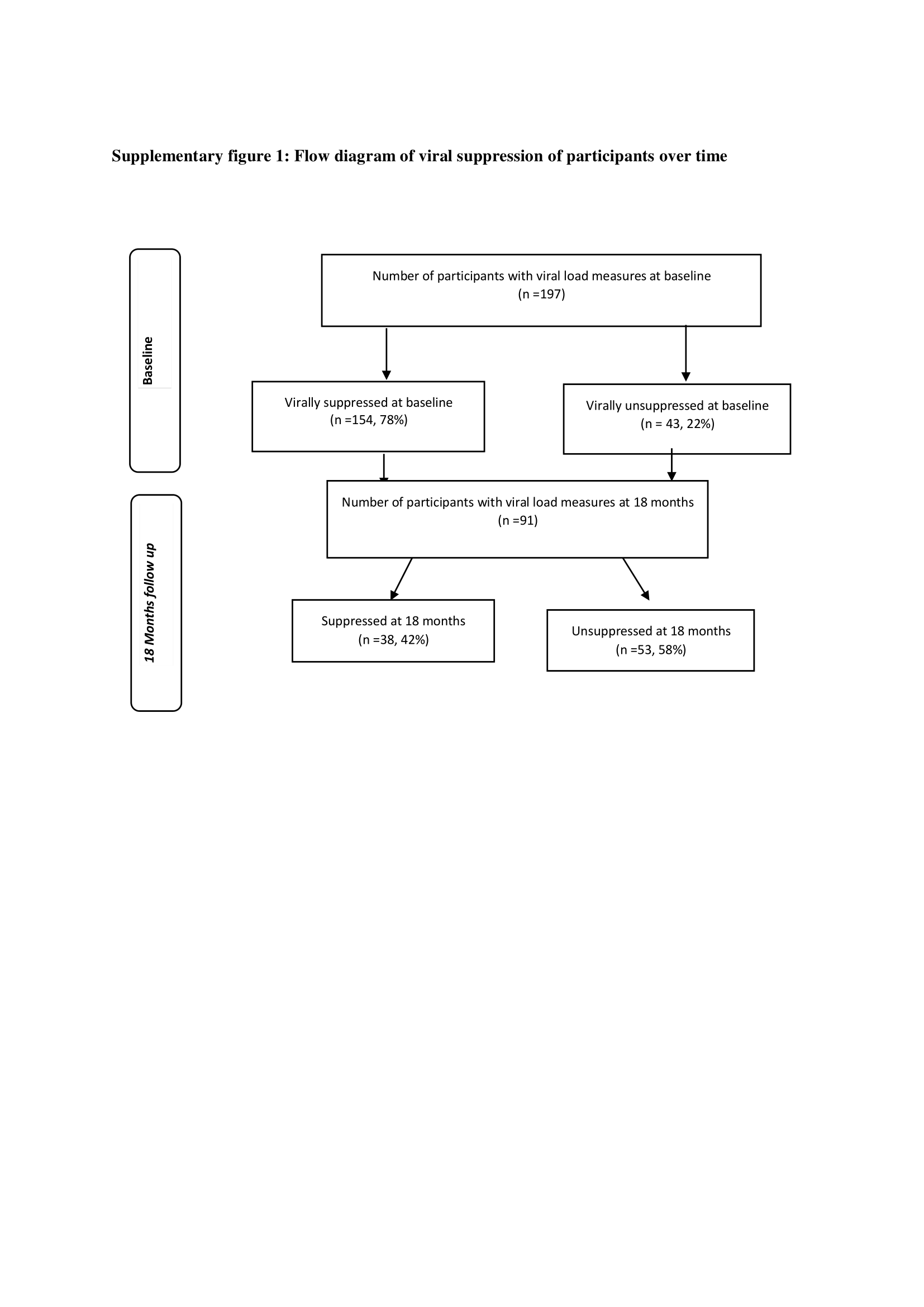

Supplement: ciz373_suppl_Supplementary_Figure_1 [file ciz373_suppl_supplementary_figure_1.png]
