# Supplementary material for: Incidence and Progression of Echocardiographic Abnormalities in Older Children with Human Immunodeficiency Virus and Adolescents Taking Antiretroviral Therapy: A Prospective Cohort Study
Source: Clin Infect Dis. 2019 May 4;70(7):1372–8. doi: 10.1093/cid/ciz373 (PMC7931829; doi:10.1093/cid/ciz373)
Supplement: ciz373_suppl_Supplementary_Table_1 [file ciz373_suppl_supplementary_table_1.docx]

**Supplementary Table 1: Antiretroviral drugs received by participants**

| **Antiretroviral drug** | **(N=175) n (%)** |
| --- | --- |
| Zidovudine | 91 (52) |
| Stavudine | 1 (1) |
| Tenofovir | 75 (43) |
| lamivudine | 173 (99) |
| Didanosine | 2 (1) |
| Abacavir | 7 (4) |
| Atazanavir | 7 (4) |
| Lopinavir/ritonavir (Aluvia) | 8 (5) |
| Atazanavir/ritonavir | 28 (16) |
| Nevirapine | 61 (35) |
| Efavirenz | 39 (22) |
