# Supplementary material for: Incidence and Progression of Echocardiographic Abnormalities in Older Children with Human Immunodeficiency Virus and Adolescents Taking Antiretroviral Therapy: A Prospective Cohort Study
Source: Clin Infect Dis. 2019 May 4;70(7):1372–8. doi: 10.1093/cid/ciz373 (PMC7931829; doi:10.1093/cid/ciz373)
Supplement: ciz373_suppl_Supplementary_Table_2 [file ciz373_suppl_supplementary_table_2.docx]

**Supplementary Table 2: Association between change in z-scores and baseline risk factors**

| Variables | RV diameter | | LV diameter | | IVS diameter | | LVPW diameter | | LA diameter | | TAPSE diameter | | |
| --- | --- | --- | --- | --- | --- | --- | --- | --- | --- | --- | --- | --- | --- |
|  | **Coefficient** | **P-value** | **Coefficient** | **P-value** | **Coefficient** | **P-value** | **Coefficient** | **P-value** | **Coefficient** | **P-value** | **Coefficient** | **P-value** |  |
| Age |  |  |  |  |  |  |  |  |  |  |  |  |  |
| 6 – 9 years | 1 | 0.78 | 1 | 0.69 | 1 | 0.80 | 1 | 0.80 | 1 | 0.26 | 1 | 0.46 |  |
| 10 -12 years | -0.080 |  | -0.071 |  | -0.004 |  | -0.089 |  | -0.167 |  | 0.006 |  |  |
| 13 – 16 years | -0.099 |  | -0.117 |  | -0.087 |  | 0.198 |  | -0.081 |  | -0.027 |  |  |
| Viral load |  |  |  |  |  |  |  |  |  |  |  |  |  |
| ≤ 400 copies/ml | 1 |  |  |  |  |  |  |  |  |  |  |  |  |
| > 400 copies/ml | -0.119 | 0.41 | 0.034 | 0.79 | 0.170 | 0.20 | 0.080 | 0.55 | 0.002 | 0.99 | -0.120 | 0.41 |  |
| CD4 count |  |  |  |  |  |  |  |  |  |  |  |  |  |
| ≥ 350 cell/μl | 1 |  |  |  |  |  |  |  |  |  |  |  |  |
| <350 cell/μl | 0.202 | 0.32 | 0.154 | 0.39 | 0.110 | 0.55 | 0.060 | 0.75 | 0.041 | 0.78 | -0.275 | 0.30 |  |
| Duration on ART |  |  |  |  |  |  |  |  |  |  |  |  |  |
| 0 – 3 years | 1 | 0.96 | 1 | 0.34 | 1 | 0.68 | 1 | 0.43 | 1 | 0.40 | 1 | 0.60 |  |
| 4 – 7 years | 0.009 |  | 0.171 |  | -0.093 |  | 0.155 |  | -0.067 |  | 0.174 |  |  |
| 8 – 12 years | 0.054 |  | 0.126 |  | 0.019 |  | 0.098 |  | 0.201 |  | 0.126 |  |  |
| Age at ART initiation |  |  |  |  |  |  |  |  |  |  |  |  |  |
| 0 -5 years | 1 | 0.07 | 1 | 0.65 | 1 | 0.80 | 1 | 0.92 | 1 | 0.75 | 1 | 0.06 |  |
| 6 – 10 years | 0.320 |  | -0.035 |  | 0.040 |  | 0.009 |  | -0.056 |  | 0.196 |  |  |
| 11 – 15 years | 0.169 |  | -0.184 |  | 0.133 |  | 0.265 |  | 0.050 |  | -0.531 |  |  |
|  |  |  |  |  |  |  |  |  |  |  |  |  |  |
| Height-for-age z-scores | -0.085 | 0.13 | -0.035 | 0.42 | 0.015 | 0.73 | -0.092 | 0.06 | -0.023 | 0.57 | -0.048 | 0.56 |  |
|  |  |  |  |  |  |  |  |  |  |  |  |  |  |
| Weight -for-age z-scores | -0.001 | 1.00 | -0.008 | 0.86 | 0.044 | 0.33 | -0.062 | 0.22 | -0.049 | 0.23 | -0.032 | 0.67 |  |

*RV, right ventricle; LV, left ventricle; IVS, interventricular septum, LVPW, left ventricular posterior wall; LA, left atrium, TAPSE, tricuspid annular plane systolic excursion*
